# Quality of life, cognitive, physical and emotional function at diagnosis predicts head and neck cancer survival: analysis of cases from the Head and Neck 5000 study

**DOI:** 10.1007/s00405-020-05850-x

**Published:** 2020-02-15

**Authors:** S. N. Rogers, A. E. Waylen, S. Thomas, C. Penfold, M. Pring, T. Waterboer, M. Pawlita, K. Hurley, A. R. Ness

**Affiliations:** 1 Evidence-Based Practice Research Centre (EPRC), Faculty of Health and Social Care, Edge Hill University, St Helens Road, Ormskirk, L39 4QP UK; 2 Regional Maxillofacial Unit, University Hospital Aintree, Liverpool, L9 1AE UK; 3 Bristol Dental School, University of Bristol, Lower Maudlin St, Bristol, BS1 2LY UK; 4 NIHR Bristol Biomedical Research Centre, University of Bristol NHS Foundation Trust and University of Bristol, Bristol, UK; 5 Infections and Cancer Epidemiology, Infection, Inflammation and Cancer Program, German Cancer Research Center (DKFZ), Heidelberg, Germany

**Keywords:** Health related quality of life, Head and neck cancer, Squamous cell carcinoma, Survival

## Abstract

**Purpose:**

The aim of this paper is to determine whether health-related quality of life (HRQOL) at diagnosis of head and neck cancer (HNC) is associated with overall survival following treatment with curative intent after adjusting for other factors.

**Methods:**

Data were collected from 5511 participants of the Head and Neck 5000 study (HN5000). HRQOL was measured using the EORTC QLQ-C30. Questionnaire and covariate data were available from 2171 participants diagnosed as follows: oral cavity (655), oropharynx HPV+ (723) and HPV− (277), and larynx (516). On average, participants were followed up 3.2 years (SD 1.2) after diagnosis. Data were adjusted for age, gender, co-morbidity, intended treatment, education level, income from benefits, smoking status and alcohol consumption.

**Results:**

There was a clinically meaningful difference between Global HRQOL scores at diagnosis and survival in an unadjusted and adjusted model: [HR = 0.86, CI 0.82–0.89, *p* < 0.001 (unadjusted) and HR = 0.90, CI 0.86–0.94, *p* < 0.001 (adjusted)]. In analyses stratified by tumour site and HPV status, this association was similarly noted before adjustment and persisted after. There were some tumour sub-site variations: improved survival for people with laryngeal cancer reporting higher levels of physical role or social functioning and people with oral cancer reporting higher levels of role or social functioning.

**Conclusion:**

As survival is the main priority for most people diagnosed with cancer, pre-treatment HRQOL is an additional factor to be included in risk stratification and case-mix adjustments. There is merit in incorporating HRQOL into routine clinical care as this is a useful facet in patient-clinician decision making, prognostication and recovery.

## Introduction

For people diagnosed with head and neck cancer (HNC) and their carers, survival is an important priority [1, 2]. Different studies have shown the importance of individual, clinical, treatment, lifestyle and social factors in predicting survival in different cancer types [3–6] but the influence of pre-treatment health-related quality of life (HRQOL) in large HNC cohorts has not previously been reported.

A meta-analysis of 30 randomised controlled trials (started between 1986 and 2004) from the European Organisation for Research and Treatment of Cancer (EORTC) included survival data for 10,108 patients with 11 different cancer sites. Although set in the context of clinical trials and not HNC specific, this study showed that baseline HRQOL gave additional prognostic information over and above that derived from sociodemographic and clinical measures [5]. These authors also reported that, for people with HNC, emotional functioning, nausea and vomiting and dyspnoea predicted survival [6].

In a systematic review of the association between HRQOL and survival in patients with HNC in 19 different studies [7], 12 studies focused on all subscales of the EORTC questionnaire and 7 focused on selected subscales. There was strong evidence for a positive association between survival and pre-treatment physical functioning and change in global QoL from pre-treatment to 6 months after treatment. These findings are at variance to other studies [8] were there appeared to be some association between selected psycho-social factors and survival, however this relationship was not strong. There is insufficient evidence for associations between survival and other pre-treatment HRQOL subscales (role functioning, emotional functioning, cognitive functioning, social functioning and mental HRQOL). Recent findings from a prospective study of 109 people with HNC [9] reported an impact of HRQOL over a longer time frame where higher levels of HRQOL at diagnosis (improved physical function and reduced sleep disturbance) predicted improved 10-year survival rates independent of clinical, individual and lifestyle factors.

Although there are studies that investigate the impact of HRQOL on survival for people diagnosed with cancer, many of them are subject to limitations: those with large sample sizes are often carried out in cohorts of people with different cancers [3–6] and those that focus on HNC are usually restricted by small samples in which it is difficult to stratify for tumour site and other factors. The UK based Head and Neck 5000 study (HN5000) [10] is a prospective study of over 5000 people diagnosed with HNC. This cohort provides a unique opportunity to explore factors at the time of diagnosis that may predict survival up to 3 years after diagnosis of HNC. The large sample size allows analyses to be stratified by tumour site and helps quantify the place of HRQOL as a predictor of survival after adjustment for other variables. The aim of this paper is to determine the effect of HRQOL in predicting overall survival for participants in the HN5000 cohort following treatment with curative intent after adjusting for other associated factors.

## Methods

Data were collected from participants in the Head and Neck 5000 prospective clinical cohort study (HN5000). Details on HN5000 have been published [10, 11] and a fully searchable data dictionary is available online (https://www.headandneck5000.org.uk/). Data were collected data at diagnosis (baseline) and 4 and 12 months and three years later using self-report questionnaires and data capture forms (DCF) to record details from clinical records. Of the 5511 people were consented into the study, 142 were subsequently found to be ineligible. The resultant study sample contained 5369 people with head and neck cancer.

### Ethics

The study was approved by the National Research Ethics Committee.

### Inclusion criteria

For this study we included people diagnosed with an oral cavity, oropharyngeal or laryngeal tumour defined using the following ICD codes: C01, C02.4, C05.1, C05.2, C05.8, C05.9, C09, C10. We excluded people who did not provide a blood sample or consent to storage of biosamples at the time of diagnosis and therefore did not have serum HPV status. We also excluded people on a palliative or supportive treatment pathway at diagnosis. This was because we expected the relationship between quality of life and survival to be different in this small group of people, compared with the majority of people who were on a curative treatment pathway.

### Questionnaire

HRQOL at diagnosis was measured using the EORTC QLQ-C30 questionnaire [12]. It comprises 30 questions combined into nine symptom scales, five functional domains and a global measure of HRQOL. For the purposes of this study we used the five functional domains (physical, role, emotional, cognitive and social functioning) and the global HRQOL as exposure variables. Scores were calculated according to EORTC guidelines [13] resulting in a range of 0–100 for each domain. Clinically meaningful differences in HRQOL were considered to be evident when there was a 10-point difference in scores.

### Outcome

The primary outcome was survival as of 1 April 2017. This was recorded via patient medical records and linkage to death certificate data through the UK Health and Social Care Information Centre (HSCIC).

### Confounders

We included various demographic, clinical and health behaviour factors that may confound the association between HRQOL and survival. These were: age at diagnosis, gender, highest educational qualification and the proportion of household income that comes from benefits; clinical tumour, node and metastatic (TNM) stage, pre-treatment co-morbidity using the overall comorbidity score from the Adult Comorbidity Evaluation (ACE)-27 [14] and intended treatment, categorised as: surgery only, surgery with adjunct therapy, chemoradiotherapy only, radiotherapy only. Health behaviours were smoking status (current, former or never smoker) and alcohol consumption. Alcohol consumption was converted into standard UK alcohol units per week [15]. We categorised this into four categories of alcohol consumption: non-drinker, moderate use, harmful use and hazardous use [16].

### Serum HPV testing

We tested blood samples for HPV status. The primary measure was serological response to HPV antibodies using a glutathione S-transferase multiplex assay carried out at the German Cancer Research Centre (DKFZ) in Heidelberg, Germany [17]. We defined seropositivity as HPV16 E6 antibodies > 1000 Median Fluorescence Intensity units (MFI) [17].

### Statistical analysis

We compared the data for participants with complete versus incomplete data. For those with complete data we stratified all analyses by tumour site, with further stratification by serum HPV status for people with oropharyngeal cancer. We described the HRQOL, demographic and clinical characteristics, and health behaviours of people in these four groups and compared them using ANOVAs (Kruskal–Wallis test for skewed data) for continuous variables and Pearson’s chi-square test for categorical variables. For the HRQOL measures we conducted further post-hoc pairwise comparisons using Dunn’s test (with Bonferroni correction) for omnibus tests where *p* < 0.1.

We used Cox proportional hazards models to investigate the effects of different patient and treatment factors on the risk of death. People alive at our latest date of follow-up were assigned as right censored at this date. We derived hazard ratios for a 10-point change in each of the QOL scales, which is considered to be a clinically meaningful difference [12] using univariable Cox regression models. We then adjusted the Cox models for age, gender, comorbidity, intended treatment, education, income from benefits, smoking status and alcohol consumption. We tested the proportional hazards assumption and found that TNM stage was not proportional. We therefore stratified our analyses by TNM stage.

To control the family wise error rate we considered a ‘family’ of statistical tests to be the Cox regression models within a specific tumour site (either unadjusted or adjusted). Using this definition, we applied a Bonferroni corrected significance level of 0.05/6 = 0.008. We considered all other results to be exploratory and significance levels for these tests were not adjusted.

## Results

**Fig. 1:**
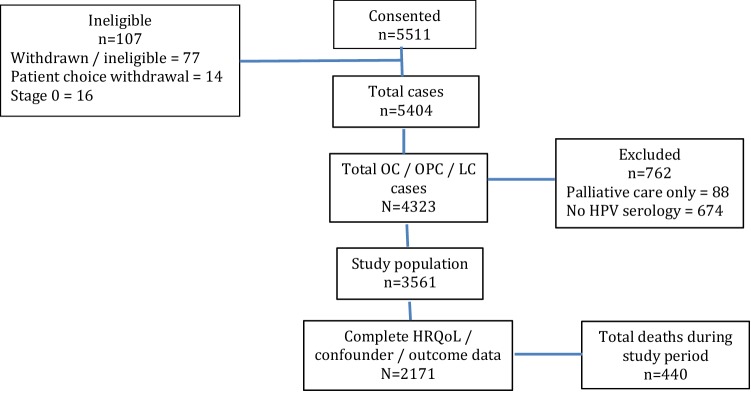
Summary of participants with oral/oropharyngeal/laryngeal cancer with complete data

From the total H&N5000 cohort confirmed as eligible to participate (*N* = 5511), 4323 people (80.5%) were diagnosed with either oral cavity (OC), oropharyngeal (OPC) or laryngeal cancer (LC). We excluded 88 people on a palliative treatment pathway and a further 674 people who did not have HPV serology available. Consequently 3561 people met our inclusion criteria and comprised our study population. We analysed data from 2171 (61.0%) participants who had complete HRQOL, confounder and outcome data (see Fig. 1); the mean follow-up time was 3.2 years (SD 1.2). There were 440 deaths during the study period with a total of 6874 person-years of follow-up.

People with complete data differed from those without complete data across most exposures and confounders (Table 1). Most notably a larger proportion of people with complete data had HPV-positive OPC tumours and were younger with fewer or less severe comorbidities. People with complete data were also more likely to have never smoked but they did report higher alcohol consumption at the time of diagnosis. Differences in HRQOL between the complete and incomplete data groups were minor, as seen by the comparable median, and upper and lower quartiles, although *p*-values were small. This reflects small differences in the relative ranking between the groups.

**Table 1:** Demographic and clinical data for people with and without complete data

	Incomplete data (*n* = 1402)	Complete data (*n* = 2171)	*p*-value
Baseline QLQ-C30	Score (25–75%)	(Score (25–75%)	
Global QoL	66.7 (50.0, 83.3)	66.7 (50.0, 83.3)	< 0.001*
Physical functioning	86.7 (66.7, 100.0)	93.3 (80.0, 100.0)	< 0.001*
Role functioning	83.3 (50.0, 100.0)	100.0 (66.7, 100.0)	< 0.001*
Emotional functioning	75.0 (58.3, 83.3)	75.0 (58.3, 91.7)	0.001*
Cognitive functioning	83.3 (66.7, 100.0)	83.3 (66.7, 100.0)	0.004*
Social functioning	83.3 (50.0, 100.0)	83.3 (66.7, 100.0)	0.016*
Age (mean (SD))	63.0 (11.0)	61.0 (10.7)	< 0.001**
Gender	*N* (%)	*N* (%)	0.035***
Male	1015 (72.7)	1647 (75.9)	
Female	381 (27.3)	524 (24.1)	
Tumour site	*N* (%)	*N* (%)	< 0.001***
Oral cavity	440 (31.5)	655 (30.2)	
Oropharynx HPV−	215 (15.4)	277 (12.8)	
Oropharynx HPV+	364 (26.1)	723 (33.3)	
Larynx	377 (27.0)	516 (23.8)	
Stage	*N* (%)	*N* (%)	0.67***
1	322 (23.2)	518 (23.9)	
2	260 (18.7)	380 (17.5)	
3	180 (13.0)	265 (12.2)	
4	627 (45.1)	1008 (46.4)	
Co-morbidity	*N* (%)	*N* (%)	< 0.001***
No co-morbidity	485 (34.7)	1013 (46.7)	
Mild discompensation	515 (36.9)	713 (32.8)	
Moderate discompensation	292 (20.9)	322 (14.8)	
Severe discompensation	81 (5.8)	74 (3.4)	
Unknown	23 (1.6)	49 (2.3)	
Treatment	*N* (%)	*N* (%)	0.005***
Surgery only	464 (33.2)	724 (33.3)	
Surgery+ adjunct	243 (17.4)	367 (16.9)	
Chemoradiation only	371 (26.6)	674 (31.0)	
Radiation only	318 (22.8)	406 (18.7)	
Education	*N* (%)	*N* (%)	< 0.001***
School level	256 (60.5)	976 (45.0)	
Further education	122 (28.8)	782 (36.0)	
University/poly	45 (10.6)	413 (19.0)	
Income from benefits	*N* (%)	*N* (%)	0.072***
None	259 (62.6)	1481 (68.2)	
Very little	40 (9.7)	192 (8.8)	
About a quarters	15 (3.6)	87 (4.0)	
About half	10 (2.4)	66 (3.0)	
About three quarters	15 (3.6)	48 (2.2)	
All	75 (18.1)	297 (13.7)	
Baseline smoking			0.008***
Current user	102 (23.1)	410 (18.9)	
Former	260 (59.0)	1237 (57.0)	
Never	79 (17.9)	524 (24.1)	
Baseline alcohol	*N* (%)	*N* (%)	< 0.001***
Non-drinker	163 (33.6)	551 (25.4)	
Moderate	115 (23.7)	503 (23.2)	
Hazardous	148 (30.5)	818 (37.7)	
Harmful	59 (12.2)	299 (13.8)	

*p-value derived from Kruskal–Wallis test

**p-value derived from ANOVA

***p-value derived from Pearson’s chi-squared test

### Variation in QoL by tumour site

There were small differences in global HRQOL and in the functional domains between people with OC, OPC (HPV±) and LC tumours at diagnosis (Table 2). People with OPC–HPV+ tumours having higher global HRQOL and higher physical functioning scores. Those with OPC–HPV + and LC tumours had higher emotional functioning scores than those with OC or OPC–HPV− tumours. People with OC had lower cognitive function scores than those with OPC–HPV+ or LC tumours and people with OPC–HPV− had lower social function scores than people with either OC or LC tumours (Table 3). In summary, people with OPC HPV+ report better HRQOL in all categories than those with tumours in other oral sites.

**Table 2:** A description of HRQOL, demographic, clinical, social and behavioural factors for different tumour sites

	Oral Cavity (*n* = 655)	Oropharynx		Larynx (*n* = 516)	*p* value
		HPV− (*n* = 277)	HPV+ (*n* = 723)		
Baseline QLQ-C30	Score (25–75%)	Score (25–75%)	Score (25–75%)	Score (25–75%)	
Global QoL	66.7 (50.0, 83.3)	66.7 (50.0, 83.3)	75.0 (58.3, 83.3)	66.7 (50.0, 83.3)	< 0.001*
Physical functioning	93.3 (80.0, 100.0)	93.3 (73.3, 100.0)	100.0 (86.7, 100.0)	93.3 (73.3, 100.0)	< 0.001*
Role functioning	100.0 (66.7, 100.0)	83.3 (66.7, 100.0)	100.0 (66.7, 100.0)	100.0 (66.7, 100.0)	0.12*
Emotional functioning	75.0 (58.3, 83.3)	66.7 (58.3, 83.3)	75.0 (66.7, 91.7)	75.0 (66.7, 91.7)	< 0.001*
Cognitive functioning	83.3 (66.7, 100.0)	83.3 (66.7, 100.0)	83.3 (66.7, 100.0)	83.3 (83.3, 100.0)	0.006*
Social functioning	83.3 (66.7, 100.0)	66.7 (50.0, 100.0)	83.3 (66.7, 100.0)	83.3 (66.7, 100.0)	0.002*
Age	61.2 (12.2)	59.5 (9.5)	58.3 (8.7)	65.3 (10.2)	< 0.001**
Gender	*N* (%)	*N* (%)	*N* (%)	*N* (%)	< 0.001***
Male	409 (62.4)	212 (76.5)	581 (80.4)	445 (86.2)	
Female	246 (37.6)	65 (23.5)	142 (19.6)	71 (13.8)	
Stage	*N* (%)	*N* (%)	*N* (%)	*N* (%)	< 0.001***
1	256 (39.1)	36 (13.0)	11 (1.5)	215 (41.7)	
2	147 (22.4)	35 (12.6)	56 (7.7)	142 (27.5)	
3	44 (6.7)	45 (16.2)	98 (13.6)	78 (15.1)	
4	208 (31.8)	161 (58.1)	558 (77.2)	81 (15.7)	
Co-morbidity	*N* (%)	*N* (%)	*N* (%)	*N* (%)	< 0.001***
No co-morbidity	293 (44.7)	114 (41.2)	404 (55.9)	202 (39.1)	
Mild discompensation	216 (33.0)	94 (33.9)	220 (30.4)	183 (35.5)	
Moderate discompensation	97 (14.8)	50 (18.1)	79 (10.9)	96 (18.6)	
Severe discompensation	30 (4.6)	14 (5.1)	8 (1.1)	22 (4.3)	
Unknown	19 (2.9)	5 (1.8)	12 (1.7)	13 (2.5)	
Treatment	*N* (%)	*N* (%)	*N* (%)	*N* (%)	< 0.001***
Surgery only	510 (77.9)	45 (16.2)	50 (6.9)	119 (23.1)	
Surgery + adjunct	98 (15.0)	56 (20.2)	168 (23.2)	45 (8.7)	
Chemorad only	25 (3.8)	135 (48.7)	438 (60.6)	76 (14.7)	
Radio only	22 (3.4)	41 (14.8)	67 (9.3)	276 (53.5)	
Education	*N* (%)	*N* (%)	*N* (%)	*N* (%)	< 0.001***
School level	279 (42.6)	116 (41.9)	291 (40.2)	290 (56.2)	
Further education	231 (35.3)	111 (40.1)	285 (39.4)	155 (30.0)	
University/poly	145 (22.1)	50 (18.1)	147 (20.3)	71 (13.8)	
Proportion of income from benefits	*N* (%)	*N* (%)	*N* (%)	*N* (%)	< 0.001***
None	440 (67.2)	163 (58.8)	554 (76.6)	324 (62.8)	
Up to half	109 (16.6)	51 (18.4)	101 (14.0)	84 (16.3)	
More than half	106 (16.2)	63 (22.7)	68 (9.4)	108 (20.9)	
Baseline smoking	*N* (%)	*N*(%)	*N* (%)	*N* (%)	< 0.001***
Current user	148 (22.6)	109 (39.4)	52 (7.2)	101 (19.6)	
Former	336 (51.3)	117 (42.2)	419 (58.0)	365 (70.7)	
Never	171 (26.1)	51 (18.4)	252 (34.9)	50 (9.7)	
Baseline alcohol	*N* (%)	*N* (%)	*N* (%)	*N* (%)	< 0.001***
Non-drinker	173 (26.4)	64 (23.1)	191 (26.4)	123 (23.8)	
Moderate	144 (22.0)	55 (19.9)	181 (25.0)	123 (23.8)	
Hazardous	238 (36.3)	94 (33.9)	281 (38.9)	205 (39.7)	
Harmful	100 (15.3)	64 (23.1)	70 (9.7)	65 (12.6)	

*p-value derived from Kruskal–Wallis test

**p-value derived from ANOVA

***p-value derived from Pearson’s chi-squared test

**Table 3:** Bonferroni corrected *p*-values from post-hoc pairwise comparisons of HRQOL by tumour site

Baseline QLQ-C30	Pairwise comparison					
	Oral cavity			Oropharynx HPV-		Oropharynx HPV+
	Oropharynx HPV−	Oropharynx HPV+	Larynx	Oropharynx HPV+	Larynx	Larynx
Global QoL	0.058	**0.014**	1.000	**< 0.001**	0.060	**0.032**
Physical functioning	0.260	**< 0.001**	0.206	**< 0.001**	1.000	**< 0.001**
Role functioning	0.059	0.394	0.737	0.672	0.551	1.000
Emotional functioning	1.000	**< 0.001**	**< 0.001**	**0.003**	**< 0.001**	0.874
Cognitive functioning	0.629	**0.014**	**0.004**	1.000	0.539	1.000
Social functioning	**0.004**	0.437	1.000	0.096	**< 0.001**	0.127

### Associations between QoL and survival

#### Global quality of life

We found a clinically meaningful difference between Global HRQOL scores at diagnosis and survival in our unadjusted model when all tumour sites were analysed together and this difference remained after adjustment: [HR = 0.86, CI 0.82–0.89, *p* < 0.001 (unadjusted) and HR = 0.90, CI 0.86–0.94, *p* < 0.001 (adjusted)] (Table 4). In analyses stratified by tumour site and HPV status this association was similarly noted before adjustment and persisted after for those with OC ([HR = 0.84, CI 0.79–0.90, *p* < 0.001 (unadjusted) and HR = 0.90, CI 0.84–0.97, *p* = 0.003 (adjusted))] or LC tumours [HR = 0.84, CI 0.78–0.90, *p* < 0.001 (unadjusted) and HR = 0.85, CI 0.76–0.87, *p* < 0.001 (adjusted)].

**Table 4:** Hazard ratios for a 10-point increase in QoL scale from unadjusted and fully adjusted Cox proportional hazards regression models—by tumour site

	All sites	Oral cavity	Oropharynx		Larynx
			HPV−	HPV+	
*N*	2169	655	277	723	515
Deaths (total)	440	170	75	81	114
Global QoL					
Unadjusted	0.86 (0.82–0.89), *p* < 0.001*	0.84 (0.79–0.90), *p* < 0.001*	0.88 (0.80–0.97), *p* = 0.01	0.93 (0.84–1.02), *p* = 0.12	0.84 (0.78–0.90), *p* < 0.001*
Fully adjusted^a^	0.90 (0.86–0.94), *p* < 0.001*	0.90 (0.84–0.97), *p* = 0.003*	0.96 (0.85–1.08), *p* = 0.47	0.97 (0.88–1.08), *p* = 0.63	0.85 (0.78–0.92), *p* < 0.001*
Physical function					
Unadjusted	0.83 (0.80–0.86), *p* < 0.001*	0.86 (0.81–0.92), *p* < 0.001*	0.87 (0.80–0.95), *p* = 0.002*	0.83 (0.75–0.91), *p* = 0.001*	0.81 (0.76–0.87), *p* < 0.001*
Fully adjusted^a^	0.88 (0.84–0.93), *p* < 0.001*	0.94 (0.86–1.02), *p* = 0.12	0.95 (0.84–1.08), *p* = 0.43	0.87 (0.76–0.99), *p* = 0.03	0.79 (0.72–0.87), *p* < 0.001*
Role function					
Unadjusted	0.91 (0.88–0.93), *p* < 0.001*	0.88 (0.84–0.92), *p* < 0.001*	0.96 (0.90–1.03), *p* = 0.29	0.95, (0.88–1.02), *p* = 0.13	0.88 (0.83–0.93), *p* < 0.001*
Fully adjusted^a^	0.92 (0.90–0.95), *p* < 0.001*	0.91 (0.86–0.96), *p* < 0.001*	1.00 (0.92–1.08), *p* = 0.97	0.94 (0.87–1.01), *p* = 0.10	0.87 (0.81–0.93), *p* < 0.001*
Emotional function					
Unadjusted	0.94 (0.91–0.98), *p* = 0.003*	0.95 (0.89–1.01), *p* = 0.12	0.94 (0.86–1.04), *p* = 0.24	1.01 (0.92–1.12), *p* = 0.80	0.93 (0.87–1.00), *p* = 0.05
Fully adjusted^a^	0.95 (0.91–0.99), *p* = 0.01	0.96 (0.90–1.03), *p* = 0.29	0.98 (0.88–1.09), *p* = 0.67	1.00 (0.90–1.12), *p* = 0.95	0.91 (0.84–0.99), *p* = 0.02
Cognitive function					
Unadjusted	0.95 (0.91–0.98), *p* = 0.004	0.95 (0.89–1.01), *p* = 0.09	0.94 (0.87–1.03), *p* = 0.19	0.97 (0.88–1.06), *p* = 0.51	0.95 (0.87–1.02), *p* = 0.17
Fully adjusted^a^	0.97 (0.93–1.01), *p* = 0.15	1.00 (0.93–1.08), *p* = 0.96	0.95 (0.85–1.05), *p* = 0.32	0.96 (0.86–1.07), *p* = 0.43	0.94 (0.86–1.02), *p* = 0.14
Social function					
Unadjusted	0.91 (0.88–0.94), *p* < 0.001*	0.89 (0.84–0.94), *p* = 0.001*	0.95 (0.88–1.02), *p* = 0.15	0.94 (0.87–1.02), *p* = 0.15	0.89 (0.84–0.95), *p* < 0.001*
Fully adjusted^a^	0.93 (0.90–0.96), *p* < 0.001*	0.92 (0.87–0.97), *p* = 0.002*	0.97 (0.89–1.05), *p* = 0.47	0.94 (0.86–1.02), *p* = 0.14	0.89 (0.83–0.96), *p* = 0.001*

Bold emphasies the significant differences

^a^Adjusted for age, gender, comorbidity, treatment intent, education, income from benefits, smoking status and alcohol consumption, and stratified by TNM stage

*p-values below Bonferroni corrected significance level (0.008)

#### Functional domains

A 10-point higher score in all but the cognitive and emotional functional domains at diagnosis was associated with improved survival when all tumour sites were analysed together (Table 4). Within the tumour sub-site analyses, global HRQOL for those with OPC tumours (irrespective of HPV status) was not associated with survival in the fully adjusted model.

Higher scores in physical (HR = 0.79, CI 0.72–0.87, *p* < 0.001) functioning were associated with improved survival in people with LC tumours. Higher scores in role [HR = 0.91, CI 0.86–0.96, *p* < 0.001 (OC); HR = 0.87, CI 0.81–0.93, *p* < 0.001 (LC)] or social functioning [HR = 0.92, CI 0.87–0.97, *p* = 0.002 (OC); HR = 0.89, CI 0.83–0.96, *p* = 0.001 (LC)] were associated with improved survival for people with either OC or LC tumours.

## Discussion

In this study we used the EORTC QLQ-C30 questionnaire to examine the association between baseline HRQOL (global score and functional domains) and survival for people from the HN5000 cohort with OC, OPC (HPV±) and LC tumours.

After adjusting for demographic and clinical confounders, our findings show that people who report higher (better) levels of HRQOL at diagnosis have a higher survival compared to those with lower self-reported HRQOL (associated with worse survival for all tumour sites). This is true for both the global HRQOL score and all functional domains except emotional and cognitive functioning. We also report tumour site-specific associations between HRQOL and survival: higher global HRQOL scores at baseline are associated with improved survival rates for those with OC and LC tumours. For people with HPV+ OPC, there was weak evidence that higher reported levels of physical functioning at baseline are associated with improved survival; however, although higher HRQOL scores are associated with survival in OC and LC tumours, HRQOL appears to be of limited prognostic value in OPC (irrespective of HPV status).

As in previous studies [3–7] our findings show that survival is worse in people who reported low levels of HRQOL (globally and in specific HRQOL domains) when their cancer was diagnosed.

### Strengths and weaknesses

This study has several strengths. First, data from the HN5000 cohort provided a large sample that enabled detailed comparison between people with different types of HNC tumours and those with OPC diagnosed as HPV± . This is an important consideration as OPC is increasing and survival differs depending on whether the tumour is HPV± [18, 19]. Second, our analyses were adjusted for a variety of relevant clinical, individual and lifestyle confounders facilitating identification of the independent impact of HRQOL, so demonstrating the potential value of baseline HRQOL as a prognostic indicator. Third, our data were collected prospectively and enabled us to report on survival three years after diagnosis. This is an important timescale for people with HNC as most cancer-related deaths are likely to have occurred by this time. Fourth, the EORTC QLQ-C30 is a commonly used and well-validated cancer-related HRQOL questionnaire [12]. Finally, participants in the HN5000 cohort are representative of standard care reflected through recruitment from a wide range of hospitals including District General Hospital Specialist centres and teaching hospitals where they received routine care rather than being recruited from clinical trials.

The study has several weaknesses. First, the response rate in the HN5000 study was satisfactory but, of those who were eligible for the study, only 61% provided complete data; we also acknowledge that people with poorer baseline HRQOL and those who were older at diagnosis were less likely to complete baseline questionnaires and therefore are under-represented in this analysis. Our complete data set also comprises a larger proportion of participants who are HPV+ than the incomplete data set—however, our findings suggest that, after comprehensive adjustment for relevant factors, HPV status has a negligible effect on the association between HRQOL and survival. Second, all of our hospitals were in the UK and so some may question the generalisability of our findings. However, we believe our data are generalisable to other countries because our demographic data are similar to smaller studies reporting HRQOL and survival [7, 20, 21].

### Implications

Pre-treatment HRQOL is an additional factor that informs risk stratification and case-mix adjustments. With increasing accessibility for patients to complete patient reported outcomes through electronic platforms it is feasible to incorporate HRQOL into routine clinical care [22]. This data can assist in patient–clinician decision making, prognostication and post-treatment recovery. Potentially by identifying patients with poorer HRQOL at baseline and being cognisant of the associated clinical characteristics, it is feasible to enhance post-treatment care and monitor more closely longitudinal HRQOL on an individual patient basis to facilitate early intervention. This could lead not only better HRQOL but also improved survival.
